# Intestinal Perforations Associated With a High Mortality and Frequent Complications During an Epidemic of Multidrug-resistant Typhoid Fever in Blantyre, Malawi

**DOI:** 10.1093/cid/ciaa405

**Published:** 2020-07-29

**Authors:** Franziska Olgemoeller, Jonathan J Waluza, Dalitso Zeka, Jillian S Gauld, Peter J Diggle, Jonathan M Read, Thomas Edwards, Chisomo L Msefula, Angeziwa Chirambo, Melita A Gordon, Emma Thomson, Robert S Heyderman, Eric Borgstein, Nicholas A Feasey

**Affiliations:** 1 Malawi Liverpool Wellcome Trust Clinical Research Programme, Blantyre, Malawi; 2 Department of Clinical Sciences, Liverpool School of Tropical Medicine, Liverpool, United Kingdom; 3 Surgical Department, College of Medicine, University of Malawi, Blantyre, Malawi; 4 Institute for Disease Modeling, Bellevue, Washington, USA; 5 Centre for Health Informatics, Computing, and Statistics, Lancaster University, Lancaster, United Kingdom; 6 Pathology Department, College of Medicine, University of Malawi, Blantyre, Malawi; 7 Institute of Infection and Global Health, University of Liverpool, Liverpool, United Kingdom; 8 University College London, London, United Kingdom; 9 Surgical Department, Ministry of Health, Queen Elizabeth Central Hospital, Blantyre, Malawi

**Keywords:** *Salmonella* Typhi, antimicrobial resistance, surgery, complication

## Abstract

**Background:**

Typhoid fever remains a major source of morbidity and mortality in low-income settings. Its most feared complication is intestinal perforation. However, due to the paucity of diagnostic facilities in typhoid-endemic settings, including microbiology, histopathology, and radiology, the etiology of intestinal perforation is frequently assumed but rarely confirmed. This poses a challenge for accurately estimating burden of disease.

**Methods:**

We recruited a prospective cohort of patients with confirmed intestinal perforation in 2016 and performed enhanced microbiological investigations (blood and tissue culture, plus tissue polymerase chain reaction [PCR] for *Salmonella* Typhi). In addition, we used a Poisson generalized linear model to estimate excess perforations attributed to the typhoid epidemic, using temporal trends in *S*. Typhi bloodstream infection and perforated abdominal viscus at Queen Elizabeth Central Hospital from 2008–2017.

**Results:**

We recruited 23 patients with intraoperative findings consistent with intestinal perforation. 50% (11/22) of patients recruited were culture or PCR positive for *S*. Typhi. Case fatality rate from typhoid-associated intestinal perforation was substantial at 18% (2/11). Our statistical model estimates that culture-confirmed cases of typhoid fever lead to an excess of 0.046 perforations per clinical typhoid fever case (95% CI, .03–.06). We therefore estimate that typhoid fever accounts for 43% of all bowel perforation during the period of enhanced surveillance.

**Conclusions:**

The morbidity and mortality associated with typhoid abdominal perforations are high. By placing clinical outcome data from a cohort in the context of longitudinal surgical registers and bacteremia data, we describe a valuable approach to adjusting estimates of the burden of typhoid fever.

Typhoid remains a major public health problem in many low- and lower-middle-income countries (LMICs), with 10.9 to 17.8 million cases estimated to occur each year [1, 2]. While most cases present with nonfocal sepsis [3], typhoid can be complicated by intestinal perforation [4]. Surgical complications of typhoid fever are well described and typically occur in the third or fourth week after onset of fever and typically arise from necrosis of Peyer’s patches in the terminal ileum [5]. Estimates of the case fatality rates of typhoid perforation remain high at 15.4% globally and a case fatality rate estimate of 20% for sub-Saharan Africa [6], with important regional differences ranging widely between 5% and 80% [7].

In cases of perforated abdominal viscus presenting in typhoid-endemic settings, the etiological agent is often assumed to be *Salmonella* Typhi; however, this is rarely confirmed because there are few diagnostic microbiology facilities in LMICs [8]. Furthermore, publicly available datasets describing longitudinal trends in abdominal perforations in LMICs are rare [4, 9–11]). Consequently, data describing “surgical typhoid” are not currently incorporated into global burden of disease (GBD) estimates of typhoid [12]. In the absence of these data, global burden estimates will underestimate the true morbidity and mortality of typhoid.

Routine, quality-assured diagnostic blood culture facilities have been available at Queen Elizabeth Central Hospital (QECH), Blantyre, Malawi, since 1998. Until 2010, *S.* Typhi was an uncommon cause of bloodstream infection (BSI). Since 2011, however, there has been a substantial increase in the number of culture-confirmed cases of typhoid at QECH, increasing from an average of 14 cases per year between 1998 and 2010, to 843 cases in 2013 [13]. Although QECH does not have the capacity to routinely identify the etiological agent responsible for perforated abdominal viscus, the Department of Surgery has systematically recorded the occurrence of macroscopic perforations identified at laparotomy since 2008.

To identify the microbial cause, and to describe morbidity and mortality of perforated abdominal viscus associated with typhoid fever in this setting, we recruited a prospective cohort of patients undergoing laparotomy for suspected intestinal perforation at QECH. Further, we placed these cases in the context of longitudinal BSI and perforation surveillance data.

## METHODS

We prospectively recruited an observational cohort of patients presenting with perforated abdominal viscus to the QECH, the largest hospital in Malawi, which serves the city and district of Blantyre and acts as a referral hospital to 13 districts in the Southern Region of Malawi. Patients undergoing laparotomy for suspected typhoid perforation or with intraoperative findings deemed by the operating surgeon to be consistent with possible typhoid perforation between February 2016 and February 2017 were eligible for inclusion. Blood cultures were taken either on admission or in the operating room and intraoperatively debrided tissue (debridement of perforated bowel edges, resected bowel, pus, lymph nodes) was retained for culture and DNA extraction. In critically ill patients unable to give consent at presentation, consent was sought postoperatively.

Microbiological samples were tested at the diagnostic microbiology laboratory of the Malawi-Liverpool-Wellcome Trust Clinical Research program (MLW). Blood samples were incubated in an aerobic BacT/Alert bottle (bioMérieux) on an automated system, and suspected *Salmonella* were identified by biochemistry. Antisera were processed as previously described [14].

Tissue from intraoperative debridement was enriched in 9 mL of buffered peptone water and cultured overnight at 37°C in air. On day 2, 2 mL of this broth was subcultured in sodium biselenite and again cultured overnight at 37°C in air. On day 3, a 10-µL loop was taken from the top of the broth and inoculated onto xylose lysine deoxycholate (XLD) agar plates and cultured overnight at 37°C in air. Suspected *Salmonella* colonies were identified by biochemistry using API 20E tests and serotyped according to the White-Kauffmann-Le Minor scheme by the following antisera: polyvalent O and H, O4, O9, Hd, Hg, Hi, Hm, and Vi antisera (Pro-Lab Diagnostics). A further 2 mL was taken from the top of the selenite broth and stored at −20°C for DNA extraction.

DNA extraction was performed from tissue selenite supernatants using the QIAamp Fast DNA Stool Mini Kit (Qiagen) pathogen-detection protocol. Elution was done using 30 μL elution buffer instead of 200 μL. Multiplex real-time polymerase chain reaction (PCR) tests were performed in a CFX96 thermal cycler (Bio-Rad) using the Quantifast Pathogen PC + IC Kit (Qiagen), targeting the pan-*Salmonella* invasion A gene, the *S.* Typhi fimbriae gene [15], and the kit’s internal control. The pan-*Salmonella*, *S*. Typhi, and internal control probes were labeled with Fluorescein amidites, Texas Red, and Victoria, respectively. A 5-minute Taq activation step at 95°C was followed by 40 cycles of annealing/extension (30 seconds, 60°C) and denaturation (15 seconds, 95°C). The PCR signals were analyzed using the CFX Manager 3.1. software (Bio-Rad) with default threshold settings. Valid PCRs required the cycle threshold signal of the internal control to range from 29 to 31. A cycle threshold less than 40 was considered to be positive in the presence of a typical exponential amplification curve. Detection of *S.* Typhi required both pan-*Salmonella* and typhoid-specific signal to be positive.

Demographic and clinical data, intraoperative findings, and outcomes were captured using OpenDataKit (https://opendatakit.org) at the time of recruitment and at the time of discharge or death. Data analysis for quantitative data was performed using STATA/SE14.1 version (StataCorp).

Retrospective data summarizing monthly counts of surgically reported intestinal perforations from January 2008 to May 2017 were collected by the Department of Surgery at QECH. In brief, all patients taken to the operating room are recorded in a log book, which is transcribed into an electronic database. Cases clearly not attributable to typhoid (ie, appendicitis, trauma, and perforated peptic ulcer) were excluded. Monthly counts of patients presenting to QECH with typhoid fever diagnosed through routine blood culture surveillance were available for the same time period [14]. We generated a generalized linear model with Poisson error distribution and an identity link to estimate excess perforations attributed to typhoid fever (Supplementary Methods). We used the fitted values of a smoothed seasonal model of monthly typhoid cases through the study period as the predictor variable. Results from this model were then used to estimate the proportion of intestinal perforations attributed to the typhoid epidemic. This analysis was implemented using R, version 3.5.1 (R Foundation for Statistical Computing) [16].

The study was approved by the Malawi College of Medicine Research and Ethics committee (COMREC P.08/14/1617).

## RESULTS

### Patients

Between March 2016 and February 2017, 24 patients undergoing laparotomy were recruited. No eligible patient declined to participate. One patient had an intraoperative finding of a gallbladder perforation and was not included in the subsequent analysis. The median age of patients was 15 years, (range 6–46 years) and 18 patients (78%) were male (Table 1).

**Table 1.** Demographics and Clinical Features of Cohort

Characteristics	Values
Demographic	
Age, median (range), years	15 (6–46)
Male, n/N (%)	18/23 (78)
Clinical symptoms or signs	
Fever prior to admission, n/N (%)	19/20 (95)
** **Duration of fever prior to admission, median (IQR), days	14 (14–21)
** **Abdominal pain, n/N (%)	23/23 (100)
** **Duration of abdominal pain, median (IQR), days	7 (4–14)
** **Vomiting, n/N (%)	10/21 (48)
** **Constipation, n/N (%)	10/23 (43)
** **Diarrhea, n/N (%)	8/22 (36)
** **Symptoms of gastrointestinal bleeding, n/N (%)	3/20 (15)
** **Jaundice, n/N (%)	1/20 (5)
** **Abdominal tenderness, n/N (%)	22/22 (100)
** **Generalized abdominal guarding, n/N (%)	16/20 (80)
** **Reduced level of consciousness, n/N (%)	2/22 (9)

Abbreviation: IQR, interquartile range.

Clinical signs and symptoms are summarized in Table 1. Data for all variables were not available for the entire cohort; therefore, denominators for these summaries ranged from 20 to 23 individuals. Fever was recorded for 20 participants; 19 of 20 (95%) reported fever prior to admission, which began a median duration of 2 weeks prior to admission (range, 2–30 days). All patients had a history of abdominal pain (median duration, 7 days; range, 2–30 days). Vomiting, constipation, or diarrhea was reported by 43%, 43%, and 35%, respectively. Three patients (14%) reported both constipation and diarrhea. Three patients (14%) presented with symptoms suggestive of gastrointestinal bleeding. Two patients (9%) presented with reduced consciousness level. On examination, most patients had a tender abdomen, and frank peritoneal signs denoted as generalized abdominal guarding were present in 80%.

Abdominal and/or chest radiograph was performed for 22 patients before undergoing surgery (in 18 patients, both investigations were done) and 13 of 22 (59%) were reported as having evidence of free gas under the diaphragm. The median time between admission and operating room was 1 day (range, 0–32 days).

### Antibiotic Treatment

All patients were treated with ceftriaxone from admission for a median of 9 days (range, 3–48 days) and metronidazole (median, 9 days; range, 3–69 days), while 14 patients received an additional course of ciprofloxacin (median, 10 days; range, 4–28 days).

### Intraoperative Findings and Surgical Treatment

**Figure 1.**
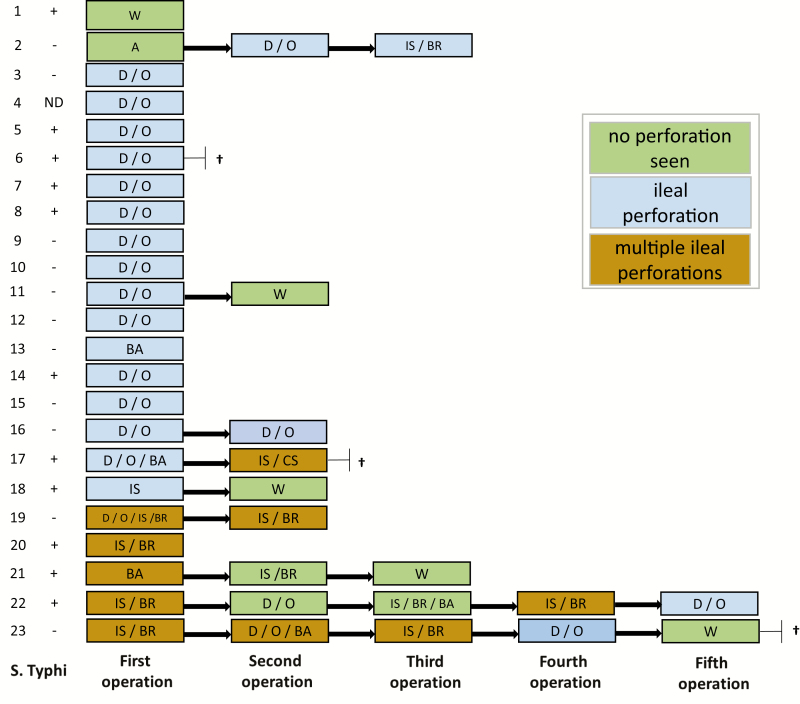
Confirmation of *Salmonella* Typhi, relating to intraoperative findings, procedures, and postoperative deaths. ^†^Patient died. Abbreviations: A, adhesiolysis; BA, bowel resection and anastomosis; CS, colostomy; D/O, debridement/oversew; IS, ileostomy; IS/BR, ileostomy with bowel resection; ND, not diagnosed; W, washout; +, confirmed by blood culture and/or tissue polymerase chain reaction; −, not confirmed.

Small-bowel perforations with a single pin hole were identified in 16 patients, while 5 patients had multiple perforations and 2 patients had no visible perforation. Intestinal perforations were all located in the ileum (summarized in Figure 1). In 1 case, the ileum was found to be inflamed without a visible perforation; there was a pelvic fluid collection and fibrinous deposits in all quadrants. This patient underwent an abdominal washout. One patient presented with a frozen abdomen with no visible intestinal injury and underwent primary adhesiolysis.

Primary debridement and oversewing of the perforation were performed in 14 of 16 (87.5%) patients with single ileal perforations. In one of these patients there was the coincidental finding of a tumor mass, prompting the fashioning of an ileocolonic anastomosis in addition to the perforation repair. One patient had a primary ileostomy performed, and 1 patient underwent bowel resection and end-to-end anastomosis. The 5 patients with multiple perforations underwent primary ileostomy and bowel resection in 4 cases, with an additional separate debridement and oversew in 1 case. One patient underwent bowel resection and end-to-end anastomosis.

Nine patients (39%) required re-laparotomy at 2 to 12 days after the initial operation (median, 4 days). Secondary perforations—all located in the ileum—were seen in 5 patients. Three of these had more than 1 secondary perforation. Bowel anastomotic leaks were seen in 7 (77%) of the 9 re-laparotomies. In 1 case, there was an isolated pus collection. Four patients underwent a third operation. Two patients underwent a total of 5 operations due to recurrent ileal perforations, anastomotic breakdowns, fluid collections, and adhesions.

### Microbiological and Molecular Confirmation of *Salmonella* Typhi

Blood cultures were taken from 14 patients on admission or on the hospital wards and *S*. Typhi was isolated from 4 patients, with 2 yielding contaminants and 8 no bacterial growth. Eleven patients had intraoperative blood cultures taken. Intraoperative tissue samples were taken from 19 patients. *Salmonella* Typhi was not isolated from any of the intraoperative blood or tissue samples; however, other Enterobacteriaceae were identified in 16 tissue samples.

Twenty-two intraoperative tissue samples from 19 patients were analyzed by multiplex PCR. *Salmonella* Typhi DNA was detected in 10 tested samples from 9 of 19 patients (47%). An additional 3 tissue samples were positive for the pan-*Salmonella* invasin A gene. Overall, 11 of 22 patients (50%) had a diagnosis of typhoid fever made by blood culture, tissue PCR, or both tests.

### Mortality and Postoperative Complications

Three of the 23 patients (13%) died. A 17-year-old male died from sepsis 2 days post–initial laparotomy. A 43-year-old male, who additionally had disseminated malignancy, died post–second laparotomy. These 2 patients had confirmed typhoid infection, representing a case fatality rate of 18% in patients with confirmed typhoid. A 17-year-old male had multiple recurrences of perforations and died after his fifth laparotomy, 6 weeks after initial admission to the hospital. This patient had a negative admission blood culture, and no intraoperative tissue was submitted in this case.

There were 4 cases of postoperative pneumonia and a further 3 cases of severe sepsis. Seven patients required admission to the intensive care unit for respiratory support. Four patients had a Bogota bag fashioned for abdominal closure either after initial surgery or after secondary surgery. Twelve patients developed wound infection, 10 of which developed wound dehiscence. Nine patients developed malnutrition despite nutritional support, and 7 were discharged on nutritional supplements. The median duration of hospital stay was 21 days (range, 4–74 days).

### Correlation of *Salmonella* Typhi Bloodstream Infections and the Intestinal Perforation Register in Queen Elizabeth Central Hospital

**Figure 2.**
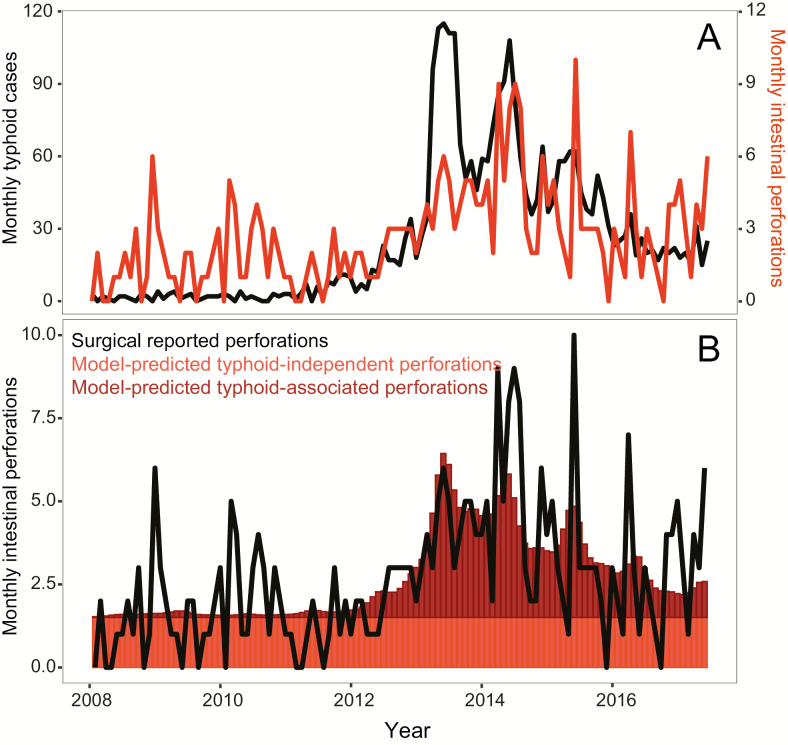
*A*, Monthly counts of intestinal perforations and typhoid cases between January 2008 and June 2015. *B*, Model-predicted surgical perforations, colored by whether the predicted perforation is typhoid independent or typhoid-associated, along with monthly reported surgical perforations.

Monthly counts of typhoid fever and intestinal perforations at QECH from January 2008 to December 2017 are shown in Figure 2A; these data are included in Supplementary Data. Results from the generalized linear model indicate that monthly case counts of *S*. Typhi are predictive of monthly intestinal perforations (*P* < .001) (Table 2). The intercept estimate of 1.5 indicates that 1.5 (95% confidence interval [CI], 1.16–1.85) intestinal perforations occur each month, independently of typhoid cases. The model estimates that, for every culture-confirmed case of typhoid, .046 (95% CI, .033–.058) perforations occur, approximately 1 perforation for every 20 culture-confirmed cases of typhoid fever presenting to QECH. Predicted intestinal perforations and their attributed causes are shown in Figure 2B. The proportion of surgical perforations predicted by typhoid fever cases is heterogeneous over time. During the recruitment period of the cohort, March 2016 to February 2017, the model independently estimates that 43% of intestinal perforations were due to typhoid fever.

**Table 2.** Intercept and Coefficient Estimates From the Generalized Linear Model Predicting Intestinal Perforations From Monthly Typhoid Cases Over the Study Period

Variable	Estimate	SE	*P*
Intercept	1.505	0.17474	<.001
Smoothed monthly typhoid cases	0.046	0.00649	<.001

## DISCUSSION

Accurate estimates of disease burden are critical to prioritize public health interventions; however, this is difficult for typhoid fever, which requires advanced diagnostics. This is particularly true of “surgical” typhoid as surgical teams do not routinely have the capacity to send blood or tissue for culture in LMICs. Furthermore, longitudinal data from surgical teams based in LMICs are rarely systematically recorded. Even when blood or tissue cultures are performed, prior antibiotic therapy and limited sensitivity frequently compromise the sensitivity of culture-based assays. As a consequence, data describing the most feared complication of typhoid fever are not represented in GBD estimates, which will lead to important underestimation of the burden of morbidity and mortality associated with typhoid fever.

In this study we have attempted to confirm infection with *S*. Typhi in cases of perforated abdominal viscus by culturing both peripheral blood and intraoperative samples, and using PCR on tissue. None of the tissue samples analyzed in our study were culture positive for *S*. Typhi by conventional microbiology. There are several possible reasons for this. *Salmonella* Typhi might simply have been outcompeted in the media by other enteric pathogens, unlike in blood which is a normally sterile site. Alternatively, as perforation is a late complication, it is possible that patients had taken antibiotics prior to presentation, rendering the samples culture negative. It has also been hypothesized that typhoid intestinal perforation may be the result of an exaggerated host response at the Peyer’s patches—the predilected site of typhoid intestinal perforation—resulting in microvascular changes, rather than a direct result of high bacterial burden [17]. We identified *S*. Typhi DNA by multiplex PCR in nearly half of the tested tissue samples. These results highlight a potential role for PCR in diagnosing surgical typhoid. In addition to the possibility of false-negative test results for *S*. Typhi, perforation may have reflected from diverse infectious and noninfectious etiologies including *Mycobacterium tuberculosis*, schistosomiasis, cytomegalovirus, malignancy, or inflammatory bowel disease. A more comprehensive suite of diagnostics including a histopathology service may have assisted in this regard.

Correlation of the longitudinal surveillance of *S*. Typhi BSI and the register of intestinal perforations at QECH showed convincing evidence that the recent surge in intestinal perforation cases coincided with the typhoid epidemic in southern Malawi. The model estimated that, although there is a baseline monthly rate of non–typhoid-attributed intestinal perforation, for every typhoid case 0.046 perforations occur. Results from the generalized linear model were consistent with observed data from the cohort; while 50% of the 22 patients recruited to the cohort were culture or PCR positive for *S*. Typhi, for the same period the model predicts that 43% of intestinal perforations were due to typhoid fever.

These results highlight the potential contribution of nonmicrobiological methods to understand the etiology of intestinal perforations. The long-term surveillance capacity for both surgical perforations and routine blood cultures, an unusual resource in this setting, has enabled this exploration. Further, the microbiological testing of surgical cases contributed an independent validation of this methodology and indicates moderate agreement.

The mortality in our cohort was substantial, with 3 deaths among 23 patients and 2 in 11 patients with confirmed infection with *S*. Typhi. Given our modeled estimates that approximately 1 in 20 cases of culture-confirmed typhoid will predict a case of intestinal perforation, and that our observed case fatality rate was 18% (consistent with other series from sub-Saharan Africa [6]), we estimate that capturing mortality due to typhoid intestinal perforation will increase the case fatality estimates of typhoid fever by 1.0% in our setting. Recent case fatality estimates for typhoid fever were 0.95% but do not factor in intestinal perforation [1]. If our findings are replicated at other sites, the inclusion of these data into GBD estimates may double mortality burden estimates for typhoid fever. The postoperative morbidity was also substantial. Nine of 23 patients required 1 or more repeated laparotomies during their illness, and several more had wound infections, pneumonia, or malnutrition. These data are also lost to GBD estimates.

Some limitations exist. This was a single-center study; however, as QECH is the only government hospital with surgical facilities in Blantyre, our data are likely to be representative of the city, although we will not have captured deaths occurring outside of the hospital from perforation, or patients seeking private care. We did not record antibiotic use prior to admission, and therefore cannot estimate the contribution to missed microbiological diagnosis. We did not have access to histopathology or tuberculosis culture. We did not perform a systematic long-term follow-up after hospital discharge and may, therefore, have underestimated morbidity and mortality. Last, although the mortality in our cohort was substantial, the cohort size was small.

We reveal an expected, but previously undescribed, burden of surgical typhoid in Blantyre, and report the associated high morbidity and mortality in the context of a general African epidemic. The systematic capture of these data may lead us to double estimates of mortality attributable to typhoid. Further data from studies of severe and complicated typhoid fever are critical to inform GBD estimates as they will support the case for widespread roll-out of typhoid conjugate vaccination.

## Supplementary Data

Supplementary materials are available at *Clinical Infectious Diseases* online. Consisting of data provided by the authors to benefit the reader, the posted materials are not copyedited and are the sole responsibility of the authors, so questions or comments should be addressed to the corresponding author.

ciaa405_suppl_Supplementary_Data

## Notes

***Acknowledgments.*** The authors thank all patients who participated in this study.

***Financial support.*** This work was supported by the Bill and Melinda Gates Foundation (grant number OPP1128444).

***Supplement sponsorship. ***This supplement is funded with support from the Coalition against Typhoid Secretariat, housed at the Sabin Vaccine Institute in Washington, DC and made possible by a grant from the Bill & Melinda Gates Foundation.

***Potential conflicts of interest.*** The authors: No reported conflicts of interest. All authors have submitted the ICMJE Form for Disclosure of Potential Conflicts of Interest.

## References

[1] Stanaway JD, Reiner RC, Blacker BF, et al The global burden of typhoid and paratyphoid fevers: a systematic analysis for the global burden of disease study 2017. Lancet Infect Dis 2019; 19:369–81. 30792131 10.1016/S1473-3099(18)30685-6 PMC6437314

[2] Antillón M, Warren JL, Crawford FW, et al The burden of typhoid fever in low- and middle-income countries: a meta-regression approach. PLoS Negl Trop Dis 2017; 11:1–21. 10.1371/journal.pntd.0005376 PMC5344533 28241011

[3] Parry CM, Hien TT, Dougan G, White NJ, Farrar JJ Typhoid fever. N Engl J Med 2002; 347:1770–82. 12456854 10.1056/NEJMra020201

[4] Butler T, Knight J, Nath SK, Speelman P, Roy SK, Azad MA Typhoid fever complicated by intestinal perforation: a persisting fatal disease requiring surgical management. Rev Infect Dis 1985; 7:244–56. 3890097 10.1093/clinids/7.2.244

[5] Bitar R, Tarpley J Intestinal perforation in typhoid fever: a historical and state-of-the-art review. Rev Infect Dis 1985; 7:257–71. 3890098 10.1093/clinids/7.2.257

[6] Mogasale V, Desai SN, Mogasale VV, Park JK, Leon Ochiai R, Wierzba TF Case fatality rate and length of hospital stay among patients with typhoid intestinal perforation in developing countries: a systematic literature review. PLoS One 2014; 9:1–11. 10.1371/journal.pone.0093784 PMC3990554 24743649

[7] Contini S Typhoid intestinal perforation in developing countries: still unavoidable deaths? World J Gastroenterol 2017; 23:1925–31. 28373758 10.3748/wjg.v23.i11.1925 PMC5360633

[8] Obaro SK, Iroh Tam PY, Mintz ED The unrecognized burden of typhoid fever. Expert Rev Vaccines 2017; 16:249–60. 27797598 10.1080/14760584.2017.1255553

[9] Ameh EA Typhoid ileal perforation in children: a scourge in developing countries. Ann Trop Paediatr 1999; 19:267–72. 10715713 10.1080/02724939992356

[10] Uba AF, Chirdan LB, Ituen AM, Mohammed AM Typhoid intestinal perforation in children: a continuing scourge in a developing country. Pediatr Surg Int 2007; 23:33–9. 17086425 10.1007/s00383-006-1796-3

[11] Bulage L, Masiira B, Ario AR, et al Modifiable risk factors for typhoid intestinal perforations during a large outbreak of typhoid fever, Kampala Uganda, 2015. BMC Infect Dis 2017; 17:1–7. 28946853 10.1186/s12879-017-2720-2 PMC5613338

[12] Qamar FN, Azmatullah A, Bhutta ZA Challenges in measuring complications and death due to invasive *Salmonella* infections. Vaccine 2015; 33:C16–20. 25921727 10.1016/j.vaccine.2015.03.103

[13] Feasey NA, Masesa C, Jassi C, et al Three epidemics of invasive multidrug-resistant *Salmonella* bloodstream infection in Blantyre, Malawi, 1998–2014. Clin Infect Dis 2015; 61:S363–71. 26449953 10.1093/cid/civ691 PMC4596930

[14] Musicha P, Cornick JE, Bar-Zeev N, et al. Trends in antimicrobial resistance in bloodstream infection isolates at a large urban hospital in Malawi (1998–2016): a surveillance study. Lancet Infect Dis 2017; 17:1042–52. 28818544 10.1016/S1473-3099(17)30394-8 PMC5610140

[15] Msefula CL, Olgemoeller F, Jambo N, et al Ascertaining the burden of invasive *Salmonella* disease in hospitalised febrile children aged under four years in Blantyre, Malawi. PLoS Negl Trop Dis 2019; 13:1–16. 10.1371/journal.pntd.0007539 PMC6663031 31314752

[16] R Core Team. A language and environment for statistical computing. Vienna, Austria: R Foundation for Statistical Computing, 2013 Available at: http://www.r-project.org. Accessed 30 August 2019.

[17] Chanh NQ, Everest P, Khoa T, et al A clinical, microbiological, and pathological study of intestinal perforation associated with typhoid fever. Clin Infect Dis 2004; 39:61–7. 15206054 10.1086/421555

